# Preparation of UV Topcoat Microcapsules and Their Effect on the Properties of UV Topcoat Paint Film

**DOI:** 10.3390/polym16101410

**Published:** 2024-05-16

**Authors:** Yongxin Xia, Xiaoxing Yan

**Affiliations:** 1 Co-Innovation Center of Efficient Processing and Utilization of Forest Resources, Nanjing Forestry University, Nanjing 210037, China; xiayongxin@njfu.edu.cn; 2 College of Furnishings and Industrial Design, Nanjing Forestry University, Nanjing 210037, China

**Keywords:** microcapsules, UV coatings, self-healing

## Abstract

An orthogonal experiment was designed to prepare different UV topcoat microcapsules by adjusting the mass ratio of wall material to core material, HLB value of emulsifier, reaction temperature, and reaction time of UV topcoat microcapsule. By testing the morphology and multiple properties of UV topcoat microcapsules, it was found that the biggest factor affecting the synthesis of UV topcoat microcapsules is the emulsifier HLB value. In order to further optimize the performance of UV topcoat microcapsules, a single-factor experiment was conducted with the emulsifier HLB value as the variable, and it was found that the UV topcoat microcapsules achieved the best performance when the emulsifier HLB value was 10.04. The optimal UV topcoat microcapsules were added to the UV topcoat at different amounts to prepare UV topcoat paint films. Through testing the various properties of the UV topcoat paint film, it was determined that the performance of the UV topcoat paint film was optimal when the amount of UV topcoat microcapsules added to the UV topcoat was 4.0%. The optical properties of the UV topcoat paint film were tested, and the effect of UV topcoat microcapsules on the color difference and glossiness of the UV topcoat paint film was not significant. The tensile and self-healing performance of UV topcoat microcapsules were tested. UV topcoat microcapsules can enhance the toughness of the UV topcoat paint film to a certain extent, suppress the generation of microcracks, and have a good self-healing effect. The results provide experimental support for the preparation of microcapsules using UV coatings as core materials for the self-healing of UV coatings.

## 1. Introduction

As a special type of coating, UV coating has the characteristics of fast curing rate and environmental protection. At the same time, the surface formed by UV coating after curing is hard and wear-resistant, with excellent chemical resistance and color stability, which can meet different needs. Therefore, it has been widely used. This includes the application of UV coatings on the surface of wooden furniture, and cured UV coatings can enhance the aesthetics and durability of wooden furniture. However, coatings are inevitably damaged by external factors during long-term use, resulting in microcracks [1,2,3,4,5,6,7]. Adding healing-type microcapsules to coatings can alleviate this problem [8,9,10,11,12].

Microcapsules are small capsule structures that contain liquid or solid materials enclosed by a wall material. The different core materials can be selected according to different application requirements, and the release effect can be controlled by adjusting the properties of the wall materials. Melamine resin is a commonly used wall material for microcapsules, which has advantages such as a simple manufacturing process, relatively low cost, good film-forming performance, and stable chemical properties. It can effectively protect its internal substances from external interference. The microcapsules using melamine resin as the wall material were successfully synthesized and applied in coatings, demonstrating the feasibility of using microcapsules in coatings [13,14,15,16,17,18]. At the same time, the results also demonstrated that melamine resin as the wall material is prone to breakage when damaged. Traditional microcapsules often use suitable substances as the core material to produce corresponding effects [19,20,21]. Considering the good properties of UV coatings, UV coatings are directly selected as the core material to prepare simple, non-toxic, and odorless melamine resin as the wall material to improve the properties of UV coatings. This has certain development prospects [22,23,24,25,26,27].

One of the important criteria for selecting core materials for self-healing microcapsules is whether natural curing can be achieved. UV coatings have the ability to quickly cure under UV light irradiation, and after curing, they have advantages such as high hardness and strong wear resistance, which meet the selection criteria for self-healing microcapsule core materials [28,29,30,31,32,33]. Therefore, the UV topcoat was selected as the core material and the melamine resin, which is simple, non-toxic, and odorless, was selected as the wall material to prepare UV topcoat microcapsules in this paper [34,35,36,37,38,39]. The orthogonal experiments and single-factor experiments were designed to optimize the preparation process of UV topcoat microcapsules, and the UV topcoat and optimal UV topcoat microcapsules were mixed to form a UV topcoat paint film. The influence of UV topcoat microcapsules on the comprehensive performance of UV topcoat film was explored.

## 2. Experimental Materials and Methods

### 2.1. Experimental Materials, Equipment, and Instruments

The materials and equipment are shown in Table 1. The size of the paint film preparation mold is 50 mm × 20 mm × 15 mm, and the material is polypropylene. The main components of UV topcoat include polyurethane acrylic resin, 1,6-hexanediol diacrylate (HDDA), photoinitiator, functional filler, extinction powder, wax powder, defoamer, dispersant, anti-settling agent, etc., and the solid content of UV topcoat is greater than 98%. The experimental instruments are detailed in Table 2, among which the lamp in the single-lamp curing machine is a UV mercury lamp with a light intensity of 80–120 W/cm^2^, and the wavelength range of ultraviolet radiation released is 250–420 nm.

### 2.2. Preparation Method of UV Topcoat Microcapsules

Based on the preliminary experiments, four factors were selected, namely the mass ratio of wall material to core material, the HLB value of emulsifier, the reaction temperature of UV topcoat microcapsule synthesis, and the reaction time of UV topcoat microcapsule synthesis, to conduct an L_9_ (3^4^) orthogonal experiment in the preparation process of UV topcoat microcapsules, in order to explore the most important factor affecting UV topcoat microcapsule synthesis. The calculation of the HLB value of the mixed emulsifier is shown in Formula (1), where *H* represents the HLB value of the mixed emulsifier, *P_T_* represents the proportion of Triton X-100 and *P_S_* represents the proportion of Span 20.

*H* = *P_T_* × 13.4 + *P_S_* × 8.6 (1)

The factors and levels of orthogonal experiments are detailed in Table 3. The arrangement of orthogonal experiments is detailed in Table 4, and the amount of materials used in orthogonal experiments is detailed in Table 5.

The preparation process of UV topcoat microcapsules mainly consists of three steps (using orthogonal experiment sample 2# as an example, where “#” represents the unit of sample number):

Wall material preparation process: the 18.02 g of 37% formaldehyde solution and 8.00 g of melamine were weighed in a beaker at a molar ratio of 3.5:1.0, and 40.0 mL of deionized water was added [40,41,42,43,44]. The solution pH was adjusted to around 9.0 using triethanolamine. The beaker was placed into a water bath, the temperature was adjusted to 60 °C, and the rotation was set to 700 rpm. The reaction proceeded for 20 min to obtain the wall material solution. It was kept warm for later use.

Core material preparation process: the 0.08 g of Triton X-100 and 0.22 g of Span 20 were weighed into a beaker, and 78.9 mL of ethanol was added. The mixture was stirred thoroughly to obtain an emulsifier solution, and then 8.80 g of UV topcoat was added. The beaker was placed into the water bath, the temperature was adjusted to 60 °C, the rotation speed was set to 700 rpm, and the reaction proceeded for 70 min to obtain the core material lotion [45,46,47,48,49].

UV topcoat microcapsule synthesis process: the wall material solution was slowly added to the core material lotion and ultrasound was applied for 15 min. Then the mixed lotion was put back into the water bath. Citric acid monohydrate was used to adjust the pH of the mixed lotion to about 4.0, and the water bath was kept at 60 °C to react for 2.0 h [50,51,52].

The reacted lotion was washed and filtered several times with ethanol and deionized water by circulating water vacuum pump after being left for 5 days. The obtained solid was put into a 60 °C oven, and the resulting powder was dried to form the UV topcoat microcapsules.

By analyzing the results of orthogonal experiments, the biggest factor affecting the synthesis of UV topcoat microcapsules was identified. A single-factor experiment was conducted using this factor as a variable to explore the optimal process for preparing UV topcoat microcapsules. The materials of single-factor experiment are detailed in Table 6.

### 2.3. Preparation Method of UV Topcoat Paint Film

The optimal UV topcoat microcapsules obtained from the single-factor experiment were added to the UV topcoat at dosages of 0%, 2.0%, 4.0%, 6.0%, 8.0%, and 10.0%. The total mass of UV topcoat microcapsules and UV topcoat was maintained at 1.50 g. Slow and uniform stirring was carried out with a glass rod, ensuring thorough mixing, and the mixture was poured into the paint film preparation mold. After the mixed topcoat had naturally flowed flat, the mold was placed on the conveyor belt of the single lamp curing machine, and the conveying speed was adjusted to 0.05 m/s. The curing time was approximately 20 s. The same total mass of mixed UV topcoat ensured that the prepared UV topcoat paint film thickness was basically the same. After the cured UV topcoat paint film was demolded, tests were conducted on optical performance, mechanical performance, and self-healing performance, with the UV topcoat paint film without UV topcoat microcapsules serving as the blank control group. Simultaneously, the mixed topcoat with different ratios mentioned above was manually brushed onto the glass plate to test the surface roughness of the UV topcoat paint film. The details of the materials used for the mixed UV topcoat microcapsules are shown in Table 7.

### 2.4. Testing and Characterization

#### 2.4.1. UV Topcoat Natural Light Curing Test

It is very important whether the core material flowing out after the rupture of self-healing microcapsules can undergo natural curing. In order to verify the feasibility of using UV topcoat directly as a microcapsule core material and whether it can cure under natural light after UV topcoat flows out, the UV topcoat was applied to the surface of the glass plate, and the curing of the UV topcoat under natural light was observed on the 1st, 4th, 7th, and 14th day, respectively, after application, and recorded.

#### 2.4.2. Yield Rate and Encapsulation Rate Testing

After the UV topcoat microcapsules obtained from the experiment had been dried until the mass no longer changed, the mass was weighed and recorded. The yield rate of the UV topcoat microcapsules was calculated using Formula (2), where *P* is the UV topcoat microcapsule yield rate, *m_p_* is the mass of the UV topcoat microcapsules produced after drying, and *m_r_* is the sum of the mass of the main components of the core and wall materials used to prepare the sample.

*P* = (*m_p_*/*m_r_*) × 100% (2)

The UV topcoat microcapsules with a mass of *m*_1_ were weighed and thoroughly ground in a mortar to destroy the structure of the UV topcoat microcapsules. The ground powder was placed into a beaker, ethanol was added, stirred, and reacted in a 70 °C water bath for 3.0 h. After the reaction was completed, deionized water and ethanol were used for multiple filtrations, and the obtained residue was placed in a 60 °C oven until the mass was constant. The dried powder was the residual wall material, and the mass was measured as *m*_2_. The encapsulation rate (*P_c_*) of the UV topcoat microcapsules was calculated using Formula (3).

*P_c_* = [(*m*_1_ − *m*_2_)/*m*_1_] × 100% (3)

#### 2.4.3. Microscopic Characterization

Optical microscope: An appropriate amount of sample was taken on a glass slide, covered with a cover glass, and placed on the optical microscope observation platform. It was adjusted to the appropriate magnification for observation.

Scanning electron microscope: An appropriate amount of sample was taken and fixed on the sample disk with double-sided adhesive tape for gold spraying. It was then placed on the observation platform inside the scanning microscope for vacuum pumping after gold spraying and adjusted to an appropriate magnification for observation.

#### 2.4.4. Chemical Composition

An appropriate amount of sample was taken and mixed evenly with a small amount of KBr powder. It was then pressed into thin sheets using a powder tablet press. The samples were tested and characterized using an infrared spectrometer.

#### 2.4.5. Optical Properties

Color difference: The color difference meter was used to test the UV topcoat paint film. The *L*, *a*, and *b* values of the UV topcoat paint film were recorded, with the test data for the control group UV topcoat paint film recorded as *L*_1_, *a*_1_, *b*_1_, and the test data for the UV topcoat paint film with UV topcoat microcapsules added recorded as *L*_2_, *a*_2_, *b*_2_. The color difference *ΔE* was calculated according to Formula (4), where Δ*L* = *L*_2_ − *L*_1_, Δ*a* = *a*_2_ − *a*_1_, Δ*b* = *b*_2_ − *b*_1_.

Δ*E* = [(Δ*L*)^2^ + (Δ*a*)^2^ + (Δ*b*)^2^] ^1/2^ (4)

Glossiness: The glossmeter was used to test the glossiness of the UV topcoat paint film at incidence angles of 20°, 60°, and 85°, in order to compare the changes in glossiness of the UV topcoat paint film with different amounts of UV topcoat microcapsules added.

Translucency: The transmittance of the UV topcoat paint film was tested using a UV spectrophotometer, with a wavelength range of 380–780 nm (visible light band) to characterize the transparency of the UV topcoat paint film with different UV topcoat microcapsule additions.

#### 2.4.6. Tensile and Roughness Testing

Elongation at break: UV topcoat paint films with different amounts of UV topcoat microcapsules were made into uniform samples and subjected to tensile testing using a universal mechanical testing machine. The elongation at break (*e*) of the UV topcoat paint film was calculated according to Formula (5), where *L*_0_ is the initial distance between the upper and lower clamping arms of the universal mechanical testing machine, and *L* is the distance between the upper and lower clamping arms when the sample fractured. The stress–strain curve of the sample was drawn using test data.

*e* = [(*L* − *L*_0_)/*L*_0_] × 100% (5)

Surface roughness: The glass plate covered with UV topcoat paint film was placed on the sample table, and the stylus of the fine roughness tester was adjusted to the surface of the UV topcoat paint film. The sample data were tested and recorded.

#### 2.4.7. Self-Healing Performance Testing

A blade was used to make marks on a glass plate covered with UV topcoat paint film, and the width of the scratch was observed and recorded under an optical microscope as *W*_1_. The width of the scratch was observed again one week later, and the width was recorded as *W*_2_. The scratch width change rate (*W*) was calculated according to Formula (6) to compare the self-healing performance of different UV topcoat paint films.

*W* = [(*W*_1_ − *W*_2_)/*W*_1_] × 100% (6)

## 3. Results and Discussion

### 3.1. Analysis of UV Topcoat Natural Light Curing Results

**Figure 1:**
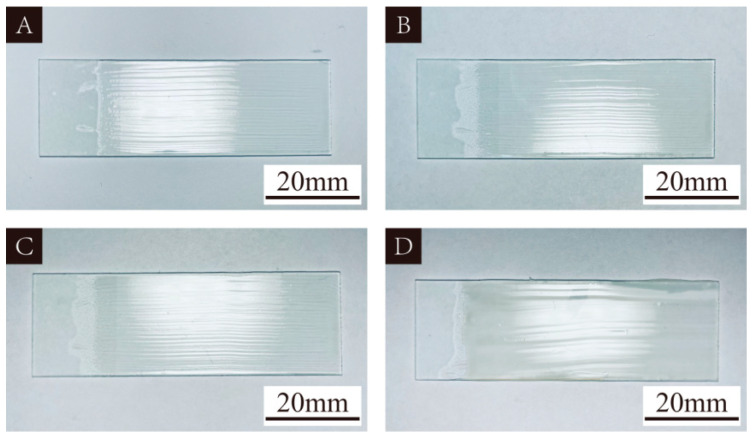
Curing process of UV topcoat under natural light: (**A**) the 1st day, (**B**) the 4th day, (**C**) the 7th day, (**D**) the 14th day.

The curing status of the UV topcoat applied to the surface of the glass plate on the 1st, 4th, 7th, and 14th day under natural light irradiation are detailed in Figure 1. It is evident from the figure that the UV topcoat is gradually curing. This is because natural light contains a certain proportion of ultraviolet light, and the irradiation of ultraviolet light gradually solidifies the UV topcoat. Although the resulting curing effect is slower compared to UV curing equipment, it does provide curing conditions and also provides strong support for the feasibility of using UV topcoat directly as a self-healing UV topcoat microcapsule core material.

### 3.2. Analysis of the UV Topcoat Microcapsules

#### 3.2.1. Analysis of UV Topcoat Microcapsule Yield Rate and Encapsulation Rate

**Figure 2:**
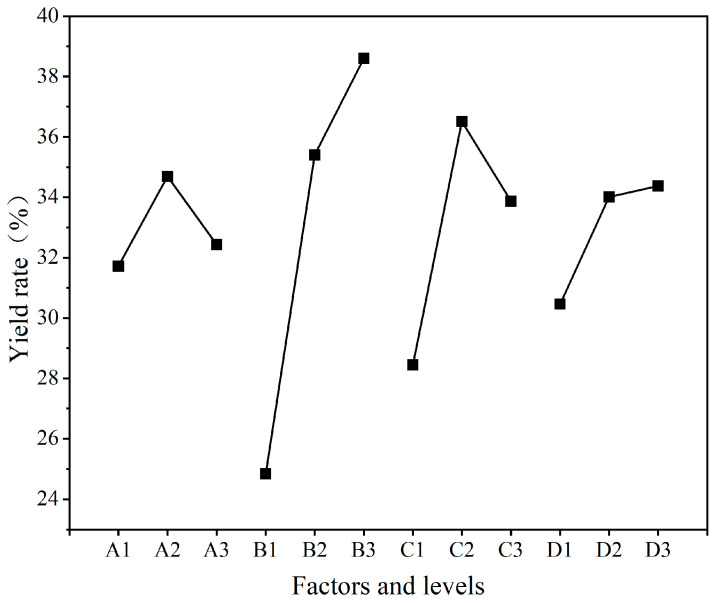
Visual analysis chart of yield rate results.

The yield rate (*P)* is one of the significant indicators to measure the success of microcapsule preparation, which is related to whether more output can be achieved under the same raw material input. It is of great significance for the efficient preparation and widespread application of microcapsules. The yield rate analysis of nine groups of UV topcoat microcapsule samples prepared by orthogonal experiments is detailed in Table 8, and the visual analysis of the yield rate results is detailed in Figure 2. Among the nine groups of UV topcoat microcapsule samples, the yield rate of sample 3# is the highest, reaching 39.72%. By comparing the size of the mean data, it can be concluded that the optimal level is A2B3C2D3. By comparing the extreme results, it can be concluded that the order of influencing factors that affect the yield rate of UV topcoat microcapsules is B > C > D > A. The most influential factor is B, which is the HLB value of the emulsifier, followed by the reaction temperature of UV topcoat microcapsule synthesis, followed by the reaction time of UV topcoat microcapsule synthesis, and finally the mass ratio of wall material to core material.

The variance analysis of yield rate results is detailed in Table 9, and it can also be concluded that factor B has the greatest impact on the yield rate results. Based on the above results, it can be concluded that factor B, namely the emulsifier HLB value, has the greatest influence on the yield rate of UV topcoat microcapsules. The recommended parameter for preparing UV topcoat microcapsules is A2B3C2D3, which is a mass ratio of wall material to core material of 1:0.7, an emulsifier HLB value of 13.40, a reaction temperature of 60 °C, and a reaction time of 3.0 h for UV topcoat microcapsule synthesis.

**Figure 3:**
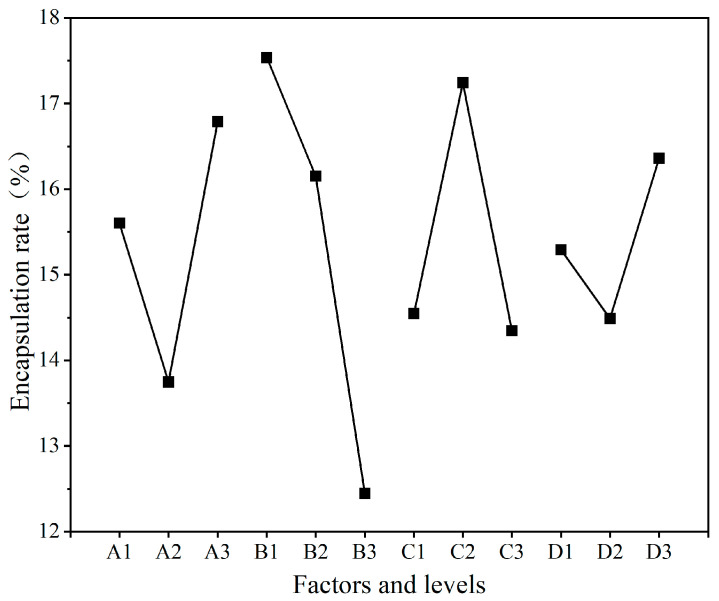
Visual analysis chart of encapsulation rate results.

The encapsulation rate (*P_c_*) is also one of the major indicators to measure the success of microcapsule preparation. It represents the ratio between the quality of the microcapsule core material and the overall quality, which can intuitively reflect the quality of the prepared microcapsules and directly affect the desired effect of the microcapsules. As for UV topcoat microcapsules, the higher the encapsulation rate, the higher the content of the core material, which means the better the self-healing effect that can be achieved in the end. The analysis of the encapsulation rate results of nine groups of UV topcoat microcapsule samples prepared by orthogonal experiments is detailed in Table 10, and the visual analysis of the encapsulation rate results is detailed in Figure 3. Among the nine groups of UV topcoat microcapsule samples, the 4# sample had the highest encapsulation rate, reaching 18.75%, followed by the 8# sample, which was 17.71%. By comparing the size of the mean data, it can be concluded that the optimal level is A3B1C2D3. By comparing the extreme results, it can be concluded that the order of influencing factors that affect the encapsulation rate of UV topcoat microcapsules is B > A > C > D. The primary influencing factor is B, namely the emulsifier HLB value.

The analysis of the variance of the encapsulation rate results, which are consistent with the range results, is detailed in Table 11. It can also be concluded that factor B has the greatest impact on the encapsulation rate results. Based on the above results, the recommended parameter for preparing UV topcoat microcapsules is A3B1C2D3, which is the mass ratio of wall material to core material of 1:0.8, an emulsifier HLB value of 8.60, a reaction temperature of 60 °C, and a reaction time of 3.0 h for UV topcoat microcapsule synthesis.

Based on the analysis of the comprehensive yield rate and encapsulation rate, it was found that factor B has the most significant influence on UV topcoat microcapsule yield rate and encapsulation rate among the four factors. Two recommended parameters, A2B3C2D3 and A3B1C2D3, were obtained simultaneously. It can be seen that the levels of C2 and D3 are consistent in the two recommended parameters. Therefore, the optimal process for preparing UV topcoat microcapsules can be determined first by determining the reaction temperature and time for UV topcoat microcapsule synthesis. Secondly, among the nine groups of UV topcoat microcapsule samples prepared by orthogonal experiments, the 4# UV topcoat microcapsule sample had the best morphology, highest encapsulation rate, and higher yield rate. Therefore, based on the preparation parameters A2B1C2D3 of the sample, the optimal process for preparing UV topcoat microcapsules can be further determined as A2B1C2D3, which means that the mass ratio of wall material to core material is 1:0.7, the HLB value of emulsifier is 8.60, and the reaction temperature for microcapsulation is 60 °C, the reaction time for microencapsulation is 3.0 h.

In order to ultimately determine the optimal process for preparing UV topcoat microcapsules, based on the superior process A2B1C2D3 mentioned above, the HLB value of the emulsifier was set as a variable, and a single-factor experiment was conducted. Based on the two recommended parameters, the HLB values of emulsifiers were 8.60 and 13.40, respectively. Therefore, in the single-factor experiment, the HLB values of emulsifiers were set to 8.60, 9.32, 10.04, 10.88, 11.72, 12.56, and 13.40, respectively.

The yield rate and encapsulation rate of seven groups of UV topcoat microcapsule samples prepared in the single-factor experiment are detailed in Table 12. It can be seen from the table that the yield rate and encapsulation rate of sample 12# are the highest, reaching 35.34% and 19.40%, respectively. Therefore, sample 12# is the optimal UV topcoat microcapsule sample, and the optimal process for preparing UV topcoat microcapsules has also been determined, namely: the mass ratio of wall material to core material is 1:0.7, the HLB value of emulsifier is 10.04, the reaction temperature for microcapsulation is 60 °C, and the reaction time for microcapsulation is 3.0 h. Therefore, in the subsequent experiments, it was chosen to add sample 12# to the UV topcoat.

#### 3.2.2. Microscopic Morphology Analysis of UV Topcoat Microcapsules

**Figure 4:**
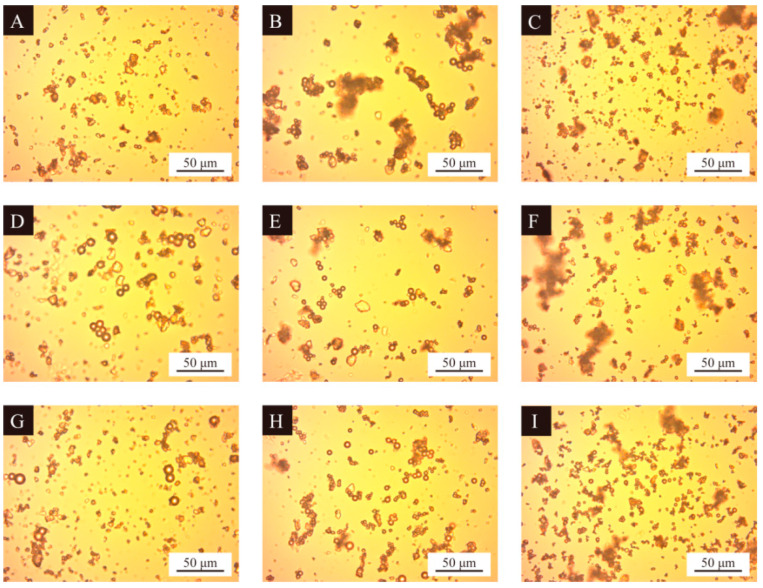
Optical microscopy images of UV topcoat microcapsule samples prepared by orthogonal experiment: (**A**–**I**) Sample 1#–9#.

The optical microscope images of nine groups of UV topcoat microcapsule samples prepared by orthogonal experiments are detailed in Figure 4. It can be observed that the UV topcoat microcapsule samples are spherical in shape and there are fewer agglomerated structures in the field of view, indicating that the UV topcoat microcapsule core material is uniformly dispersed and completely coated by the wall material. Among them, the morphology of samples 4#, 5#, and 8# is better, with uniform particle size, and less aggregation. The morphology of samples 3#, 6#, and 9# is poor, with few UV topcoat microcapsules and severe aggregation. This may be because the emulsifier HLB value of these three samples is too high, which affects the dispersion of the core material in the lotion, thus affecting the utilization of raw materials and the synthesis of UV topcoat microcapsules.

**Figure 5:**
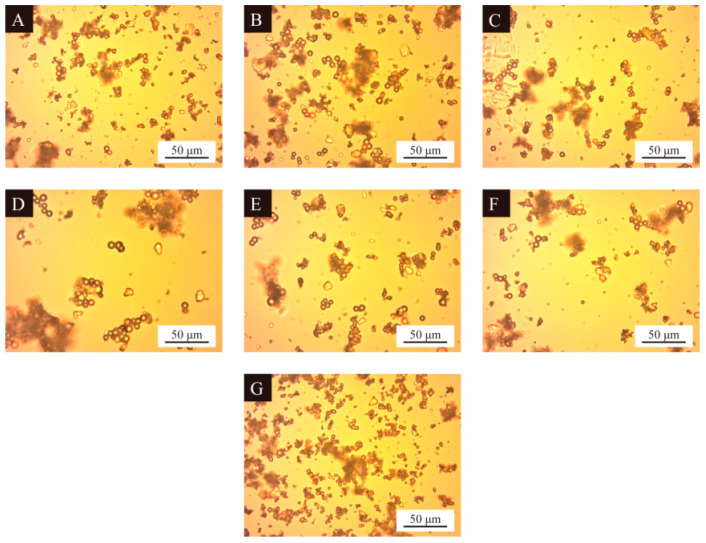
Optical microscopy images of UV topcoat microcapsule samples prepared by single-factor experiment: (**A**–**G**) Sample 10#–16#.

The optical microscope images of seven groups of UV topcoat microcapsule samples prepared by single-factor experiments are detailed in Figure 5. The morphology of samples 12# and 13# is good and relatively dispersed. The HLB values of emulsifiers were 10.04 and 10.88, respectively, indicating that neutral emulsifier HLB value is more conducive to the synthesis and dispersion of UV topcoat microcapsules. Samples 11# and 16# exhibit severe aggregation, with emulsifier HLB values of 9.32 and 13.40, respectively. This indicates that emulsifiers that are too lipophilic or hydrophilic can lead to aggregation, which is not conducive to the synthesis and dispersion of UV topcoat microcapsules. Therefore, a neutral emulsifier HLB value is more suitable for the preparation of UV topcoat microcapsules.

**Figure 6:**
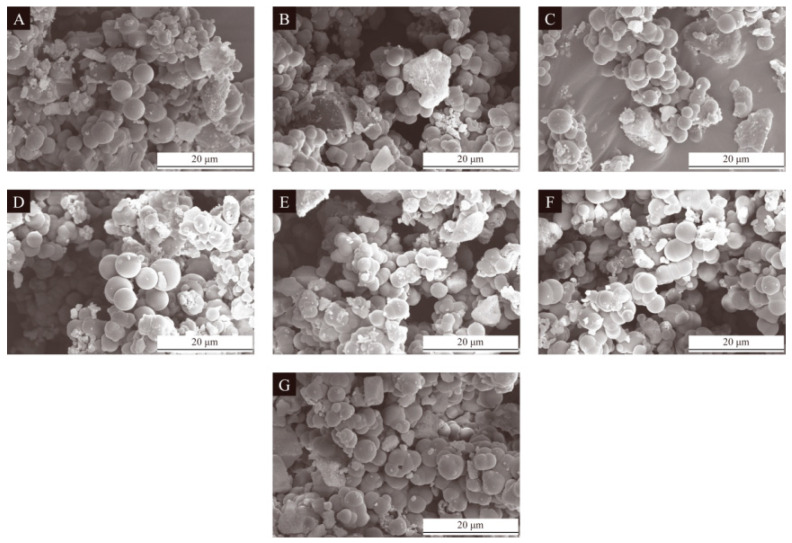
SEM images of UV topcoat microcapsule samples prepared by single-factor experiment: (**A**–**G**) Sample 10#–16#.

The scanning electron microscopy images of the seven groups of UV topcoat microcapsule samples prepared by the single-factor experiment are detailed in Figure 6. From Figure 6A,B, it can be seen that when the HLB value of the emulsifier is relatively small, the number of UV topcoat microcapsules is small and there are surface irregularities. From Figure 6C,D, it can be seen that when the HLB value of the emulsifier is relatively neutral, the number of UV topcoat microcapsules increases, the surface is smooth, and the agglomeration phenomenon is also relatively mild. From Figure 6E–G, it can be seen that as the HLB value of the emulsifier continues to increase, there are more UV topcoat microcapsules but severe aggregation and adhesion between UV topcoat microcapsules occur. This is because the main component of the UV topcoat used in this paper, such as polyurethane acrylic resin, has a certain hydrophilicity due to the presence of polyurethane chain segments. For example, propylene glycol diacrylate and hexanediol diacrylate are both diacrylate compounds, and due to the presence of hydroxyl functional groups, they also have certain hydrophilicity. Other components in UV topcoats, such as wax powder, are usually hydrophobic and have strong lipophilicity. Therefore, high or low HLB values of emulsifiers cannot disperse the core material UV topcoat well, resulting in more agglomeration and adhesion between UV topcoat microcapsules.

Based on the comprehensive analysis of UV topcoat microcapsule yield rate, encapsulation rate, and microstructure, sample 12# has the highest yield rate and encapsulation rate, the best morphology, uniform dispersion, and less agglomeration. Therefore, the UV topcoat microcapsules of sample 12# were chosen to add into the UV topcoat at 0%, 2.0%, 4.0%, 6.0%, 8.0%, and 10.0%, respectively, to prepare the UV topcoat paint films, in order to investigate the effect of UV topcoat microcapsules on the optical properties, tensile resistance, roughness, and self-healing performance of the UV topcoat paint film.

#### 3.2.3. Analysis of Chemical Composition

**Figure 7:**
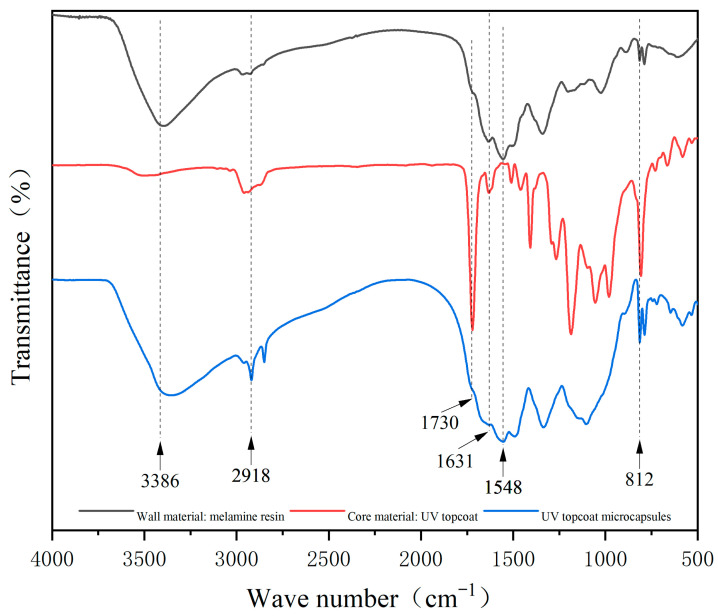
Infrared spectrum of UV topcoat microcapsules.

The infrared spectrum of UV topcoat microcapsules is detailed in Figure 7. The absorption peak at 1548 cm^−1^ was the NH bending vibration peak, and the triazine ring bending vibration absorption peak appeared at 812 cm^−1^. These are characteristic peaks of melamine resin, which appeared in the absorption curves of the wall material and UV topcoat microcapsules, indicating the successful preparation of the wall material melamine resin and the presence of the chemical composition of melamine resin in the UV topcoat microcapsules. The absorption peaks at 1631 cm^−1^, 1730 cm^−1^, and 2918 cm^−1^ are C=C, C=O, and C-H stretching vibration peaks, respectively. These are common characteristic peaks of polyurethane acrylic resin, propylene glycol diacrylate, and hexanediol diacrylate, which are the main components of the core material UV topcoat. They appeared in the absorption curves of the core material and UV topcoat microcapsules, indicating the presence of chemical components of the UV topcoat in the UV topcoat microcapsules. The above characteristic peaks demonstrate the successful encapsulation of UV topcoat microcapsules.

### 3.3. Analysis of UV Topcoat Paint Film Performance

#### 3.3.1. Analysis of UV Topcoat Paint Film Morphology

**Figure 8:**
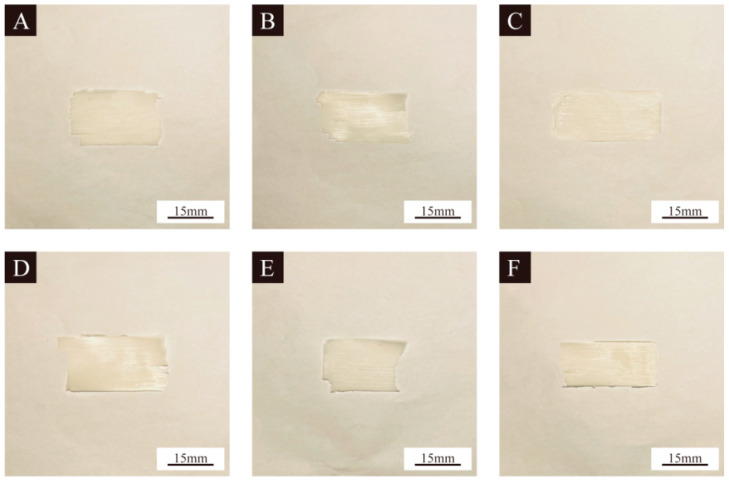
Macro morphology of UV topcoat paint film with different content of UV topcoat microcapsules added: (**A**–**F**) 0%, 2.0%, 4.0%, 6.0%, 8.0%, and 10.0%, respectively.

**Figure 9:**
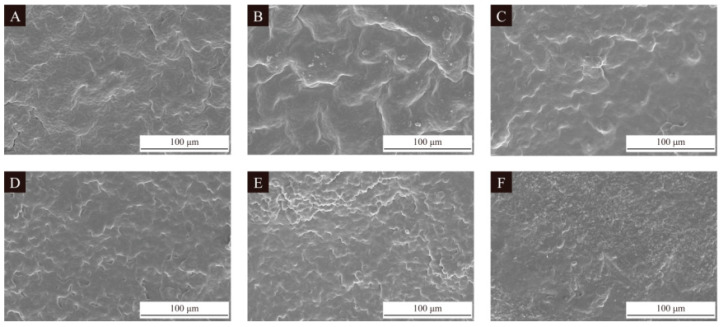
SEM images of UV topcoat paint films with different content of UV topcoat microcapsules added: (**A**–**F**) 0%, 2.0%, 4.0%, 6.0%, 8.0%, and 10.0%, respectively.

The morphology of the UV topcoat paint film with the addition of 0%, 2.0%, 4.0%, 6.0%, 8.0%, and 10.0%, respectively, UV topcoat microcapsules is detailed in Figure 8. From the figure, it can be observed that as the content of UV topcoat microcapsules increases, the color of the UV topcoat paint film gradually turns white. This is because the UV topcoat microcapsules themselves are white. As the content of UV topcoat microcapsules rises, the distribution of UV topcoat microcapsules in the UV topcoat paint film becomes wider, causing the color of the UV topcoat paint film to gradually turn white. However, the overall difference in the UV topcoat paint film is not significant, so further analysis needs to be carried out from SEM images of the UV topcoat paint film. The SEM images of the UV topcoat paint film with 0%, 2.0%, 4.0%, 6.0%, 8.0%, and 10.0% UV topcoat microcapsules, respectively, added are detailed in Figure 9. It can be seen that when the amount of UV topcoat microcapsules added is less than 6.0%, the overall surface of the UV topcoat paint film is relatively flat and smooth. When too many UV topcoat microcapsules are added, the surface of the UV topcoat paint film becomes uneven. This is because the proportion of UV topcoat microcapsules in the UV topcoat paint film is too high, resulting in uneven dispersion of UV topcoat microcapsules, which affects the surface smoothness of the UV topcoat paint film. It can be concluded that UV topcoat microcapsule content higher than 6.0% has a significant negative impact on the smoothness of the UV topcoat paint film and the dispersion of UV topcoat microcapsules in the UV topcoat paint film.

#### 3.3.2. Chemical Composition Analysis of UV Topcoat Paint Film

**Figure 10:**
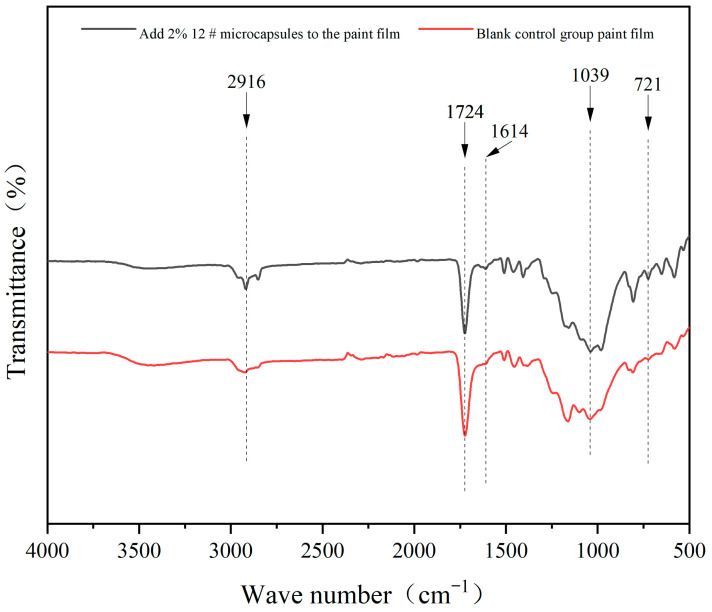
Infrared spectrum of UV topcoat paint films.

The infrared spectrum of the UV topcoat paint film with added UV topcoat microcapsules and the blank control group UV topcoat paint film is detailed in Figure 10. A triazine ring bending vibration absorption peak appeared at 721 cm^−1^, which is a characteristic peak of melamine resin. It appeared in the absorption curve of the UV topcoat paint film with added UV topcoat microcapsules but was almost invisible in the absorption curve of the blank control group UV topcoat paint film, indicating that the wall material exists in the UV topcoat paint film with added UV topcoat microcapsules and not in the blank control group UV topcoat paint film. The absorption peaks at 1614 cm^−1^, 1724 cm^−1^, and 2916 cm^−1^ are C=C, C=O, and C-H stretching vibration peaks, respectively. These are common characteristic peaks of the main components of UV topcoat, polyurethane acrylic resin, propylene glycol diacrylate, and hexanediol diacrylate. They appeared in both absorption curves, indicating that the chemical composition of the UV topcoat is not damaged after mixing with UV topcoat microcapsules. The presence of these characteristic peaks indicates that the chemical composition of both UV topcoat and UV topcoat microcapsules remains intact.

#### 3.3.3. Analysis of Optical Properties of UV Topcoat Paint Films

**Figure 11:**
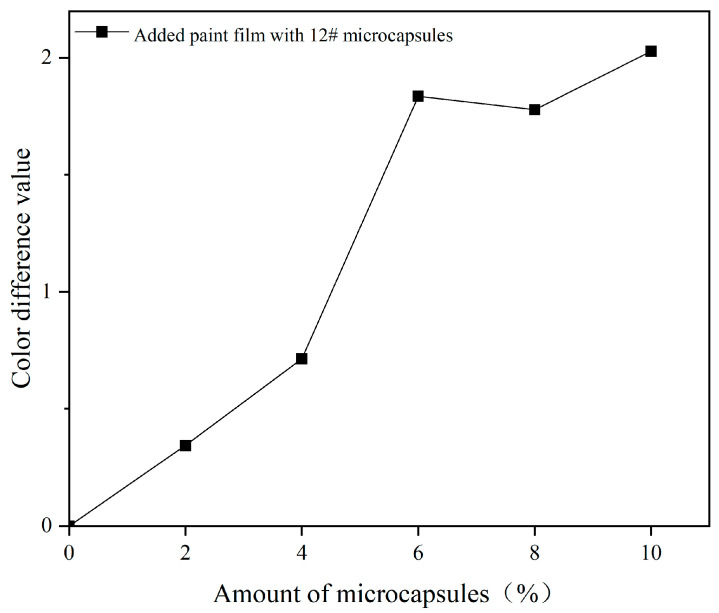
The effect of different amounts of UV topcoat microcapsules added on the color difference of UV topcoat paint films.

The effects of UV topcoat microcapsules on the color difference and chromaticity value of UV topcoat paint films under different amounts are detailed in Figure 11 and Table 13. As the content of UV topcoat microcapsules increases, the color difference of the UV topcoat paint film with UV topcoat microcapsules added relative to the blank UV topcoat paint film continues to increase, showing an upward trend, with a maximum value of 2.03. The overall *L*-value shows a downward trend, indicating that the brightness of the UV topcoat paint film is gradually decreasing. This is because UV topcoat microcapsules are granular powders, which affect the fluidity of the paint itself after being added to the coating, resulting in a decrease in the surface smoothness of the cured UV topcoat paint film, and thus a gradual decrease in brightness.

**Figure 12:**
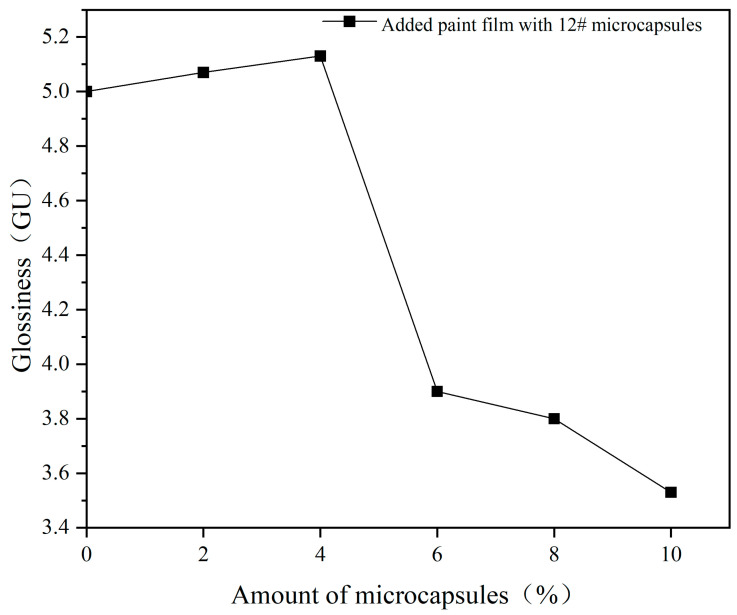
The effect of different amounts of UV topcoat microcapsules on the glossiness of UV topcoat paint films at a 60° incidence angle.

The effect of different amounts of UV topcoat microcapsules on the glossiness of UV topcoat paint films is detailed in Figure 12 and Table 14. As the amount of UV topcoat microcapsules added increases, the glossiness of the UV topcoat paint film shows a decreasing trend. When the amount reaches 6.0%, the glossiness sharply decreases. This is because the UV topcoat microcapsules affect the smoothness of the cured UV topcoat paint film. When the amount is too high, the surface of the UV topcoat paint film becomes rough, which enhances the diffuse reflection of light and leads to a decrease in glossiness.

**Figure 13:**
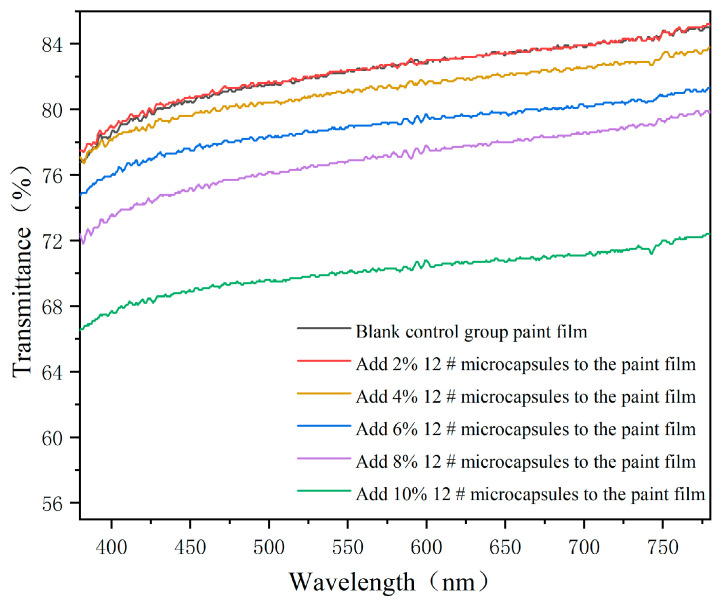
The transmittance of UV topcoat paint films with different amounts of UV topcoat microcapsules added.

The effect of different amounts of UV topcoat microcapsules on the transmittance of UV topcoat paint films is detailed in Figure 13. With the increase in UV topcoat microcapsules, the transmittance of the UV topcoat paint film gradually decreases. This is because UV topcoat microcapsules are white and opaque, and adding them to the UV topcoat will affect the transmittance. From the graph, it can be clearly seen that in the band of 380–780 nm, the transmittance of the UV topcoat paint film with UV topcoat microcapsules added is lower than that without UV topcoat microcapsules added, indicating that the addition of UV topcoat microcapsules has an impact on the transmittance of the UV topcoat paint film throughout the entire visible light wavelength, which is consistent with the macroscopic changes in the UV topcoat paint film.

#### 3.3.4. Analysis of Tensile Resistance and Roughness of UV Topcoat Paint Film

**Figure 14:**
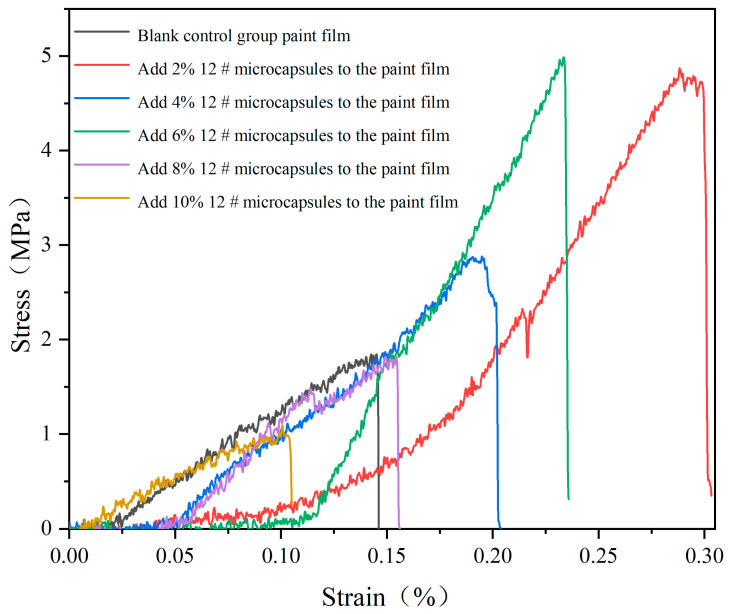
The tensile resistance of UV topcoat paint films with different amounts of UV topcoat microcapsules added.

The effect of different amounts of UV topcoat microcapsules on the tensile resistance of UV topcoat paint films is detailed in Figure 14. It can be seen that when the content of UV topcoat microcapsules is below 6.0%, the UV topcoat paint film has a certain elastic region, and overall, it shows a decreasing trend with the increase in content. This is because the distribution of granular UV topcoat microcapsules reduces the ductility of UV topcoat. When the amount of UV topcoat microcapsule added continues to increase, the elastic region of the film suddenly decreases, and the ductility of the film decreases. This is because too many UV topcoat microcapsules strengthen the hardness of the UV topcoat paint film, but also make the UV topcoat paint film more brittle.

The fracture elongation of the UV topcoat paint film under different UV topcoat microcapsule amounts is detailed in Table 15. The fracture elongation first increases and then decreases with the increase in the amount. The elongation at break of the UV topcoat paint film without UV topcoat microcapsules is 0.66%. When the amount is 4.0%, the elongation at break reaches the highest of 1.50% and then begins to decrease. This is because when the amount of UV topcoat microcapsules added is small, they can be evenly distributed within the UV topcoat paint film, thereby enhancing the toughness of the UV topcoat paint film and increasing its elongation at break. When the content of UV topcoat microcapsules is too high, the excessive amount of UV topcoat microcapsule particles significantly reduces the viscosity and elasticity of the coating itself. Therefore, when subjected to tensile force, the relative tensile strength of the UV topcoat paint film decreases, and the elongation at break decreases.

The surface roughness (R_a_) of the UV topcoat paint film under different amounts of UV topcoat microcapsules added is detailed in Table 16. As the amount of UV topcoat microcapsules added increases, the roughness of the UV topcoat paint film increases. This is because the prepared UV topcoat microcapsules are spherical particles, which still exist in a granular form after curing with UV topcoat. With the continuous increase in UV topcoat microcapsule content, the number of particles increases, resulting in an uneven surface of the UV topcoat paint film and an increase in roughness.

#### 3.3.5. Analysis of Self-Healing Performance of UV Topcoat Paint Film

**Figure 15:**
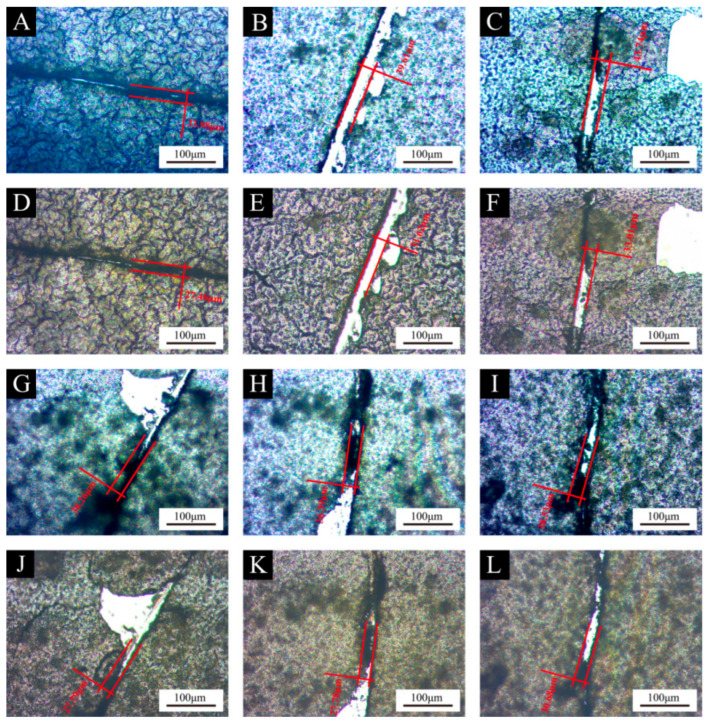
Comparison of scratches before and after self-healing of UV topcoat paint films with different amounts of UV topcoat microcapsules added: (**A**) before self-healing with 0% addition, (**B**) before self-healing with 2.0% addition, (**C**) before self-healing with 4.0% addition, (**D**) after self-healing with 0% addition, (**E**) after self-healing with 2.0% addition, (**F**) after self-healing with 4.0% addition, (**G**) before self-healing with 6.0% addition, (**H**) before self-healing with 8.0% addition, (**I**) before self-healing with 10.0% addition, (**J**) after self-healing with 6.0% addition, (**K**) after self-healing with 8.0% addition, and (**L**) after self-healing with 10.0% addition.

The self-healing performance of the UV topcoat paint film under different UV topcoat microcapsule amounts is detailed in Figure 15 and Table 17. As the content of UV topcoat microcapsules increases, the self-healing rate of UV topcoat paint film increases first and then decreases. When no UV topcoat microcapsules were added, the self-healing rate of the UV topcoat paint film was 19.19%. When the amount of UV topcoat microcapsules added was 6%, the self-healing rate of the UV topcoat paint film reached its maximum, which was 26.89%, an increase of 7.70% compared to the blank control group sample. When the content of UV topcoat microcapsules continues to rise, the self-healing rate of the UV topcoat paint film begins to decrease but remains higher than that of the blank control group sample. This is because when the content of UV topcoat microcapsules is small, UV topcoat microcapsules can be well dispersed in the UV topcoat paint film. When the UV topcoat paint film is broken, the core material in the UV topcoat microcapsules flows out, and the core material itself is a UV topcoat. It gradually solidifies under natural light irradiation, making the microcracks bond. When the content of UV topcoat microcapsules is too high, the UV topcoat microcapsule gradually agglomerates in the UV topcoat paint film, which makes the core material difficult to flow out and causes the toughness of the UV topcoat paint film to decline, resulting in the overall self-healing rate of the UV topcoat paint film.

## 4. Conclusions

The optimal preparation process of UV topcoat microcapsules through orthogonal experiments and single-factor experiments was explored, and the yield rate, encapsulation rate, morphology, particle size, and chemical composition of UV topcoat microcapsules were analyzed. The best UV topcoat microcapsule sample prepared was added to the UV topcoat to prepare a UV topcoat paint film. The morphology, chemical composition, optical properties, tensile resistance, roughness, and self-healing performance of the UV topcoat paint film were tested and explored. The optimal preparation process for UV topcoat microcapsules is as follows: the mass ratio of wall material to core material is 1:0.7, the HLB value of the emulsifier is 10.04, the reaction temperature is 60 °C, and the reaction time is 3.0 h. Based on the yield rate, encapsulation rate, and microstructure of UV topcoat microcapsules, sample 12# UV topcoat microcapsule prepared under the parameter of emulsifier HLB value of 10.04 in the single-factor experiment were the best sample, with the highest yield rate and encapsulation rate, reaching 35.34% and 19.40%, respectively. They were evenly dispersed, with smooth and round particles and the best morphology. Based on the comprehensive test results of the UV topcoat paint film, when the amount of UV topcoat microcapsules added is 4.0%, the comprehensive performance of the UV topcoat paint film is the best. At this time, the color difference of the UV topcoat paint film is 0.71, the glossiness at a 60° incidence angle is 5.13 GU, the elongation at break is 1.50%, and the roughness is 1.631 μm. The self-healing rate is 24.53%. The results provide practical support for the application of UV microcapsules in UV coatings and lay the foundation for subsequent research on UV microcapsules.

## Figures and Tables

**Table 1:** Experimental materials.

Experimental Materials	Molecular Mass (g/mol)	CAS	Producer
37% formaldehyde	30.03	50-00-0	Shandong Xinjiuchenghuagong Technology Co., Ltd., Jinan, China
Melamine	126.12	108-78-1	Jiangning District Wanjuyi Experimental Equipment Firm, Nanjing, China
Triethanolamine	149.19	102-71-6	Nanjing Houxin Biotechnology Co., Ltd., Nanjing, China
Span-20	346.459	133-39-2	Nanjing Houxin Biotechnology Co., Ltd., Nanjing, China
Triton X-100	646.85	9002-93-1	Shandong Yousuo Chemical Technology Co., Ltd., Linyi, China
Absolute ethanol	46.07	64-17-5	Wuxi Jingke Chemical Co., Ltd., Wuxi, China
UV topcoat	-	-	Jiangsu Himonia Technology Co., Ltd., Zhenjiang, China
Citric acid monohydrate	210.139	5949-29-1	Jinan Xiaoshi Chemical Co., Ltd., Jinan, China
Paint film preparation mold	-	-	Dongguan Xinchen Industrial Investment Co., Ltd., Dongguan, China

**Table 2:** Experimental instruments.

Experimental Instruments	Model	Manufacturer
Water bath	LC-OB-5L	Hunan Yunyihui E-commerce Co., Ltd., Changsha, China
Scanning electron microscope	OLS3000	Jiangdong Jiecheng Electronic Components Store, Ningbo, China
Powder tablet press	HY-12	Tianjin Tianguang Optical Instrument Co., Ltd., Tianjin, China
Infrared spectrometer	Cary630	Shenyang Jasco Trading Co., Ltd., Shenyang, China
Color difference instrument	3nhYS3010	Shenzhen threenh Technology Co., Ltd., Shenzhen, China
Glossmeter	3nhYG60S	Shenzhen threenh Technology Co., Ltd., Shenzhen, China
Ultraviolet spectrophotometer	U-3900	Hitachi High-Tech Co., Ltd., Beijing, China
Universal mechanical testing machine	5000N	Zhejiang Wanxiong Instrument Manufacturing Co., Ltd., Ningbo, China
Coating roughness tester	SJ-411	Dongguan Asktools Co., Ltd., Dongguan, China
Single-lamp curing machine	620#	Huzhou Tongxu Machinery Equipment Co., Ltd., Huzhou, China

**Table 3:** Orthogonal experimental factors and levels.

Level	Factor A m_wall material_:m_core material_	Factor B HLB Value of Emulsifier	Factor C Temperature (°C)	Factor D Time (h)
1	1:0.6	8.60	50	1.0
2	1:0.7	10.04	60	2.0
3	1:0.8	13.40	70	3.0

**Table 4:** Orthogonal experiment schedule.

Sample (#)	Factor A m_wall material_:m_core material_	Factor B HLB Value of Emulsifier	Factor C Temperature (°C)	Factor D Time (h)
1	1:0.6	8.60	50	1.0
2	1:0.6	10.04	60	2.0
3	1:0.6	13.40	70	3.0
4	1:0.7	8.60	60	3.0
5	1:0.7	10.04	70	1.0
6	1:0.7	13.40	50	2.0
7	1:0.8	8.60	70	2.0
8	1:0.8	10.04	50	3.0
9	1:0.8	13.40	60	1.0

**Table 5:** Materials for orthogonal experiment.

Sample (#)	Triton X-100 (g)	Span 20 (g)	Ethanol (mL)	UV Topcoat (g)	Formaldehyde (g)	Melamine (g)	Deionized Water (mL)
1	0.00	0.30	78.90	8.80	18.02	8.00	40.00
2	0.08	0.22	78.90	8.80	18.02	8.00	40.00
3	0.30	0.00	78.90	8.80	18.02	8.00	40.00
4	0.00	0.30	78.90	8.80	15.44	6.86	35.00
5	0.08	0.22	78.90	8.80	15.44	6.86	35.00
6	0.30	0.00	78.90	8.80	15.44	6.86	35.00
7	0.00	0.30	78.90	8.80	13.52	6.00	30.00
8	0.08	0.22	78.90	8.80	13.52	6.00	30.00
9	0.30	0.00	78.90	8.80	13.52	6.00	30.00

**Table 6:** Materials table of single-factor experiment.

Sample (#)	Triton X-100 (g)	Span 20 (g)	Ethanol (mL)	UV Topcoat (g)	Formaldehyde (g)	Melamine (g)	Deionized Water (mL)
10	0.00	0.30	78.90	8.80	15.44	6.86	35.00
11	0.05	0.25	78.90	8.80	15.44	6.86	35.00
12	0.08	0.22	78.90	8.80	15.44	6.86	35.00
13	0.15	0.15	78.90	8.80	15.44	6.86	35.00
14	0.20	0.10	78.90	8.80	15.44	6.86	35.00
15	0.25	0.05	78.90	8.80	15.44	6.86	35.00
16	0.30	0.00	78.90	8.80	15.44	6.86	35.00

**Table 7:** Materials table of blend topcoat.

Amount of UV Topcoat Microcapsules (%)	Mass of UV Topcoat Microcapsules (g)	Mass of UV Topcoat (g)
0	0	1.50
2.0	0.03	1.47
4.0	0.06	1.44
6.0	0.09	1.41
8.0	0.12	1.38
10.0	0.15	1.35

**Table 8:** Analysis of UV topcoat microcapsule yield rate results.

Sample (#)	Factor A m_wall material_:m_core material_	Factor B HLB Value of Emulsifier	Factor C Temperature (°C)	Factor D Time (h)	*P* (%)
1	1:0.6	8.60	50	1.0	16.63
2	1:0.6	10.04	60	2.0	38.80
3	1:0.6	13.40	70	3.0	39.72
4	1:0.7	8.60	60	3.0	31.58
5	1:0.7	10.04	70	1.0	35.59
6	1:0.7	13.40	50	2.0	36.91
7	1:0.8	8.60	70	2.0	26.31
8	1:0.8	10.04	50	3.0	31.81
9	1:0.8	13.40	60	1.0	39.16
Mean value 1	31.717	24.840	28.450	30.460	
Mean value 2	34.693	35.400	36.513	34.007	
Mean value 3	32.427	38.597	33.873	34.370	
Range	2.976	13.4057	8.063	3.910	
Order of influencing factors	B > C > D > A				
Optimal level	A2	B3	C2	D3	
Optimal scheme	A2B3C2D3				

**Table 9:** Analysis of variance of yield rate results.

Sources of Variation	Quadratic Sum	Free Degree	F-Ratio	F-Critical Value	Significance
Factor A	14.502	2	0.128	4.460	
Factor B	310.978	2	2.735	4.460	
Factor C	101.399	2	0.892	4.460	
Factor D	27.999	2	0.246	4.460	
Error	454.88	8			

**Table 10:** Analysis of UV topcoat microcapsule encapsulation rate results.

Sample (#)	Factor A m_wall material_:m_core material_	Factor B HLB Value of Emulsifier	Factor C Temperature (°C)	Factor D Time (h)	*P_c_* (%)
1	1:0.6	8.60	50	1.0	16.84
2	1:0.6	10.04	60	2.0	17.35
3	1:0.6	13.40	70	3.0	12.62
4	1:0.7	8.60	60	3.0	18.75
5	1:0.7	10.04	70	1.0	13.40
6	1:0.7	13.40	50	2.0	9.09
7	1:0.8	8.60	70	2.0	17.02
8	1:0.8	10.04	50	3.0	17.71
9	1:0.8	13.40	60	1.0	15.63
Mean value 1	15.603	17.537	14.547	15.290	
Mean value 2	13.4047	16.153	17.243	14.487	
Mean value 3	16.787	12.447	14.347	16.360	
Range	3.040	5.090	2.896	1.873	
Order of influencing factors	B > A > C > D				
Optimal level	A3	B1	C2	D3	
Optimal scheme	A3B1C2D3				

**Table 11:** Analysis of variance of encapsulation rate results.

Sources of Variation	Quadratic Sum	Free Degree	F-Ratio	F-Critical Value	Significance
Factor A	14.089	2	0.735	4.460	
Factor B	41.561	2	2.169	4.460	
Factor C	15.703	2	0.819	4.460	
Factor D	5.300	2	0.277	4.460	
Error	76.65	8			

**Table 12:** UV topcoat microcapsule yield rate and encapsulation rate of single-factor experiment.

Sample (#)	Factor B HLB Value of Emulsifier	*P* (%)	*P_c_* (%)
10	8.60	31.70	18.70
11	9.32	30.84	17.80
12	10.04	35.34	19.40
13	10.88	31.48	18.20
14	11.72	30.42	19.30
15	12.56	29.32	18.80
16	13.40	30.96	14.00

**Table 13:** The chromaticity and color difference values of UV topcoat paint films with different content of UV topcoat microcapsules added.

Amount of 12# UV Topcoat Microcapsules (%)	*L*	*a*	*b*	Δ*E*
0	76.53	0.50	1.43	-
2.0	76.47	0.53	2.00	0.34
4.0	74.93	0.23	2.57	0.71
6.0	75.20	0.27	2.73	1.84
8.0	75.10	0.23	2.97	1.78
10.0	76.53	0.50	1.43	2.03

**Table 14:** The glossiness of UV topcoat paint films with different amounts of UV topcoat microcapsules added.

Amount of 12# UV Topcoat Microcapsules (%)	Glossiness at 20° (GU)	Glossiness at 60° (GU)	Glossiness at 85° (GU)
0	1.17	5.00	3.60
2.0	0.87	5.07	2.83
4.0	1.27	5.13	5.67
6.0	0.90	3.90	3.17
8.0	1.00	3.80	7.27
10.0	0.87	3.53	7.73

**Table 15:** Elongation at break of UV topcoat paint film with different amounts of UV topcoat microcapsules added.

Amount of 12# UV Topcoat Microcapsules (%)	Elongation at Break (%)
0	0.66
2.0	1.22
4.0	1.50
6.0	1.14
8.0	0.62
10.0	0.41

**Table 16:** Roughness of UV topcoat paint films with different amounts of UV topcoat microcapsules added.

Amount of 12# UV Topcoat Microcapsules (%)	R_a_ (μm)
0	0.369
2.0	1.590
4.0	1.631
6.0	2.598
8.0	2.698
10.0	3.706

**Table 17:** Self-healing rate of UV topcoat paint films with different amounts of UV topcoat microcapsules added.

Amount of 12# UV Topcoat Microcapsules (%)	Scratch Width (μm)		Self-Healing Rate (%)
	After Scratching	One Week Later	
0	33.98	27.46	19.19
2.0	39.61	31.62	20.16
4.0	43.74	33.01	24.53
6.0	38.26	27.97	26.89
8.0	36.39	27.78	23.67
10.0	38.53	30.68	20.38

## Data Availability

Data are contained within the article.
